# Supplementary material for: The use of digital texture image analysis in determining the masticatory efficiency outcome
Source: PLoS One. 2021 May 6;16(5):e0250936. doi: 10.1371/journal.pone.0250936 (PMC8101913; doi:10.1371/journal.pone.0250936)

S1 Values for DTIA and VOH variables

Fig 1

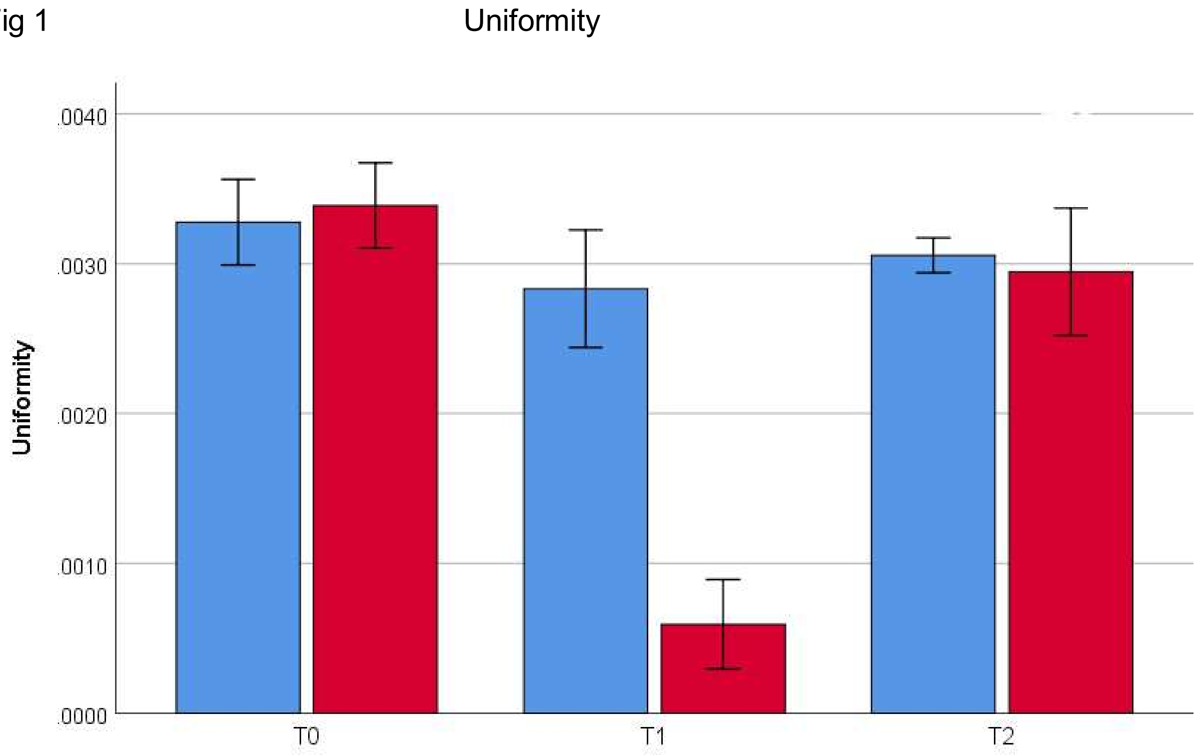

Fig 2

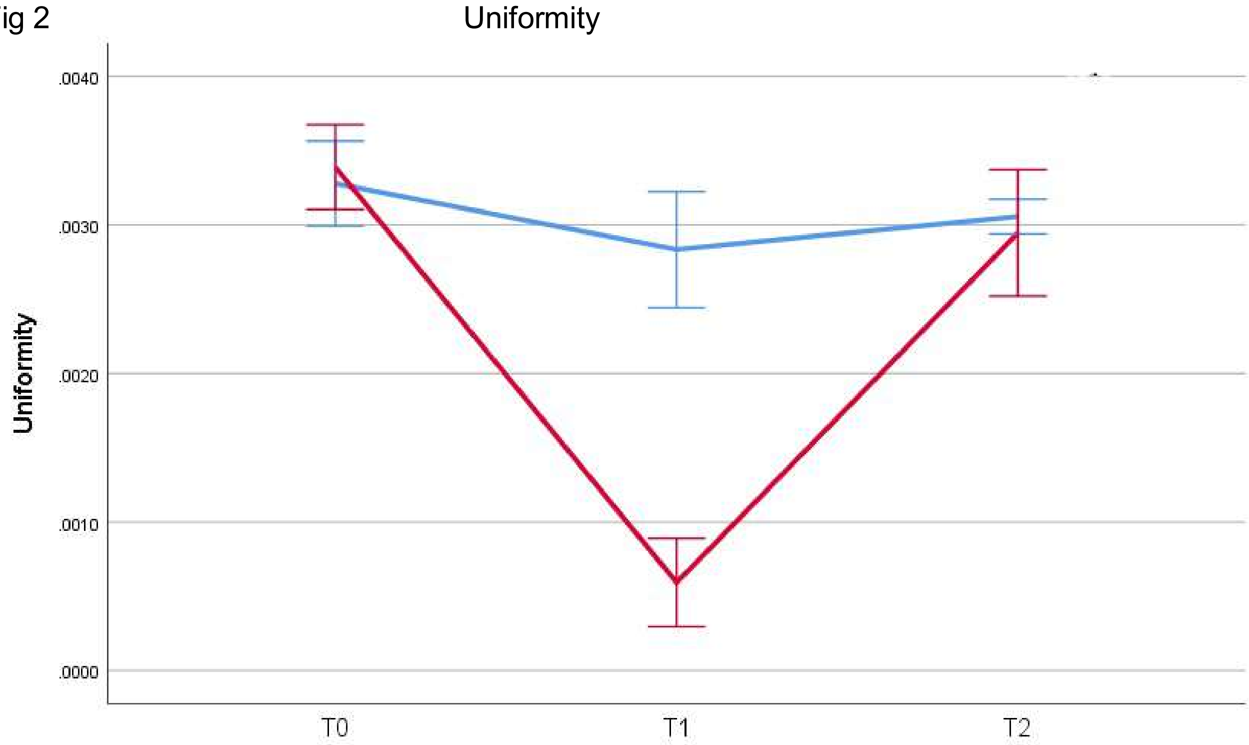

Fig 3 Contrast

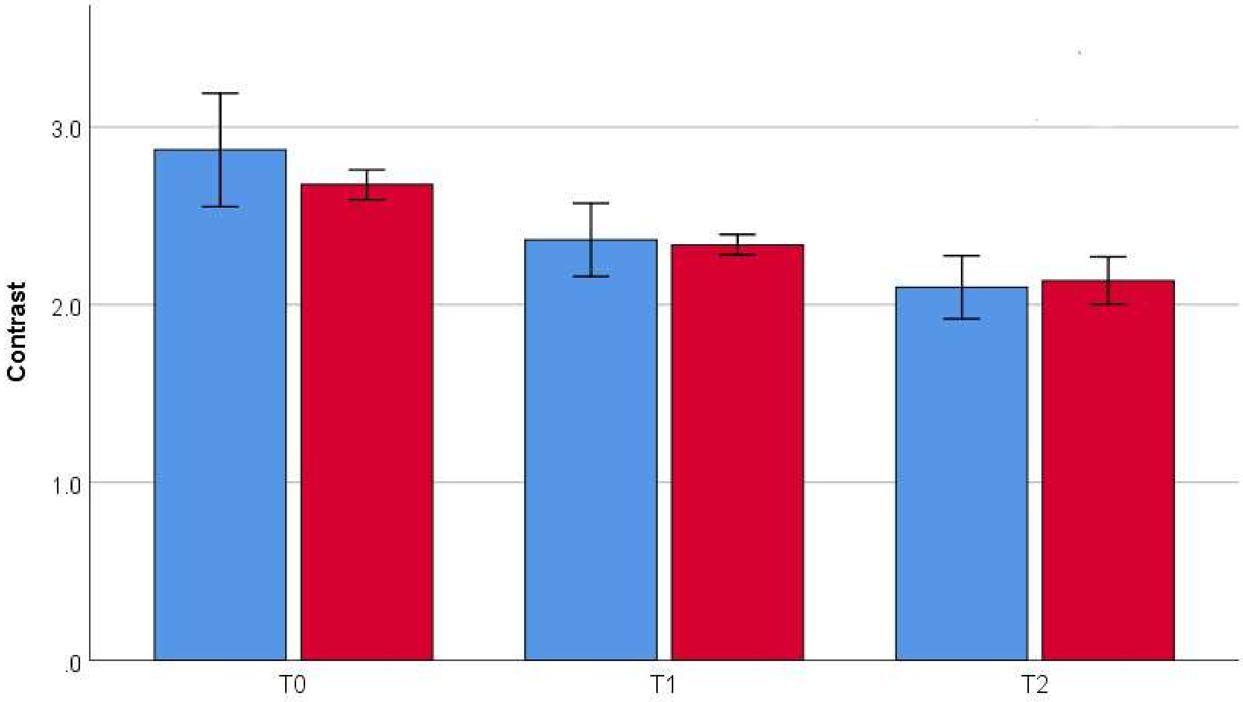

Fig 4 Contrast

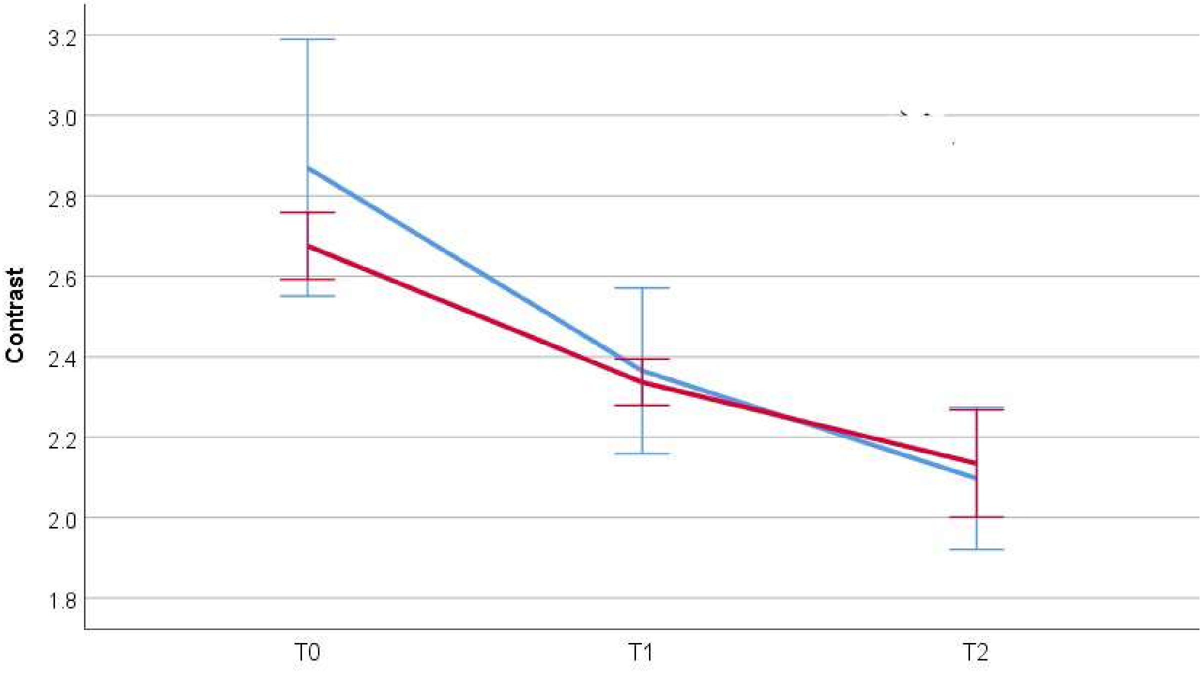

Fig 5

Homogenity

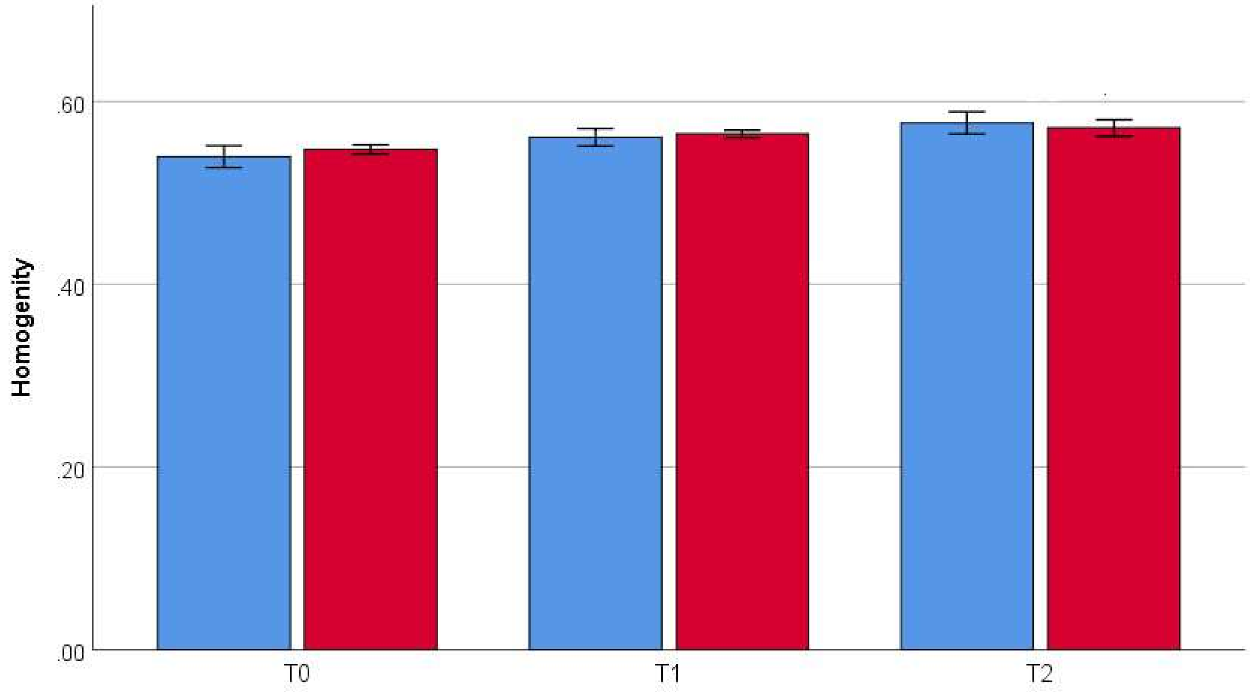

Fig 6

Homogenity

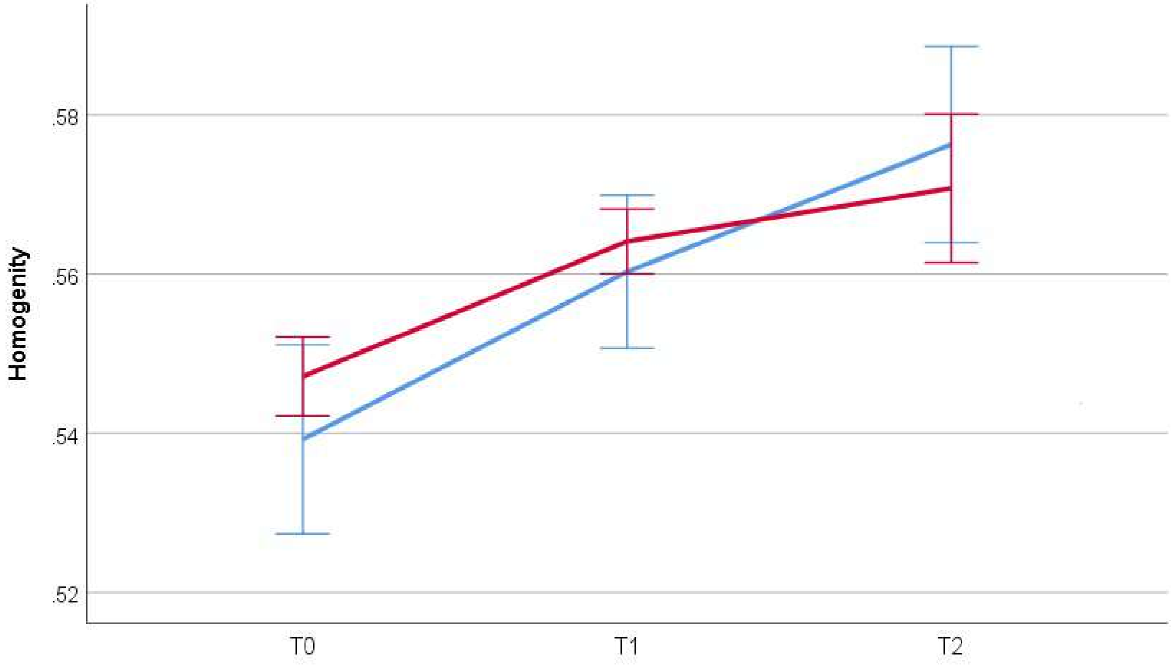

Fig 7

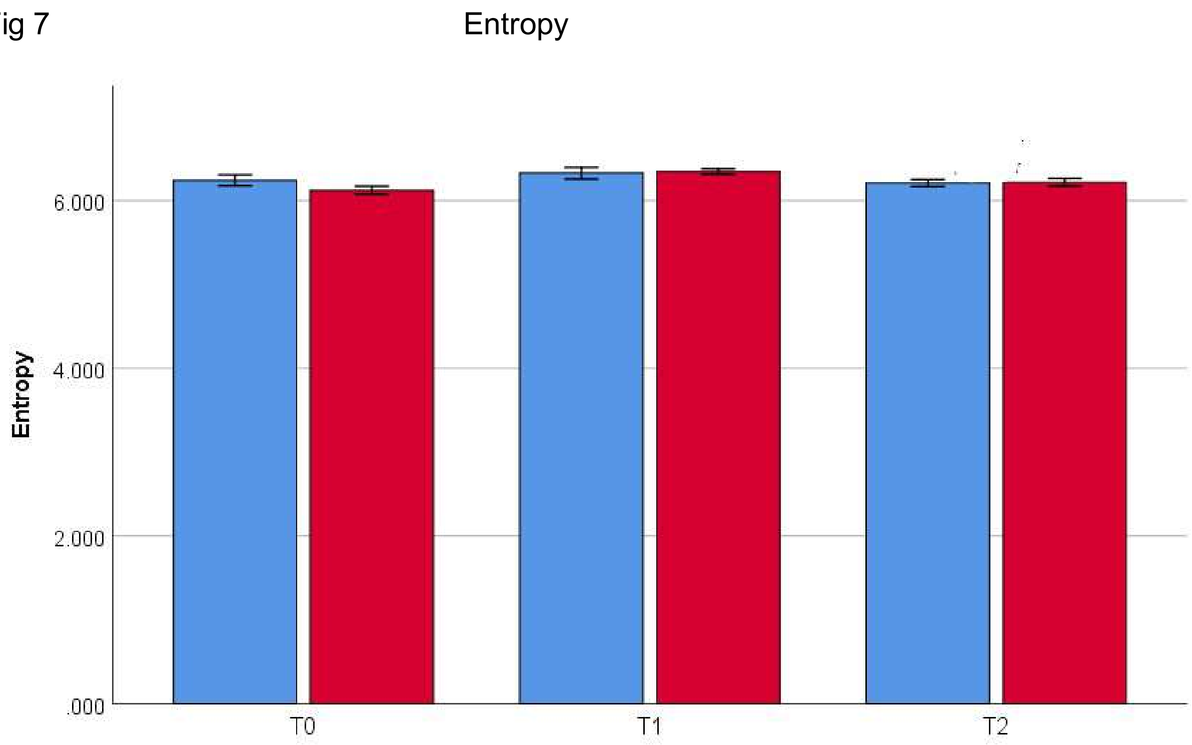
Entropy
Entropy
6.000
4.000
2.000
.000
T0
T1
T2

Fig 8

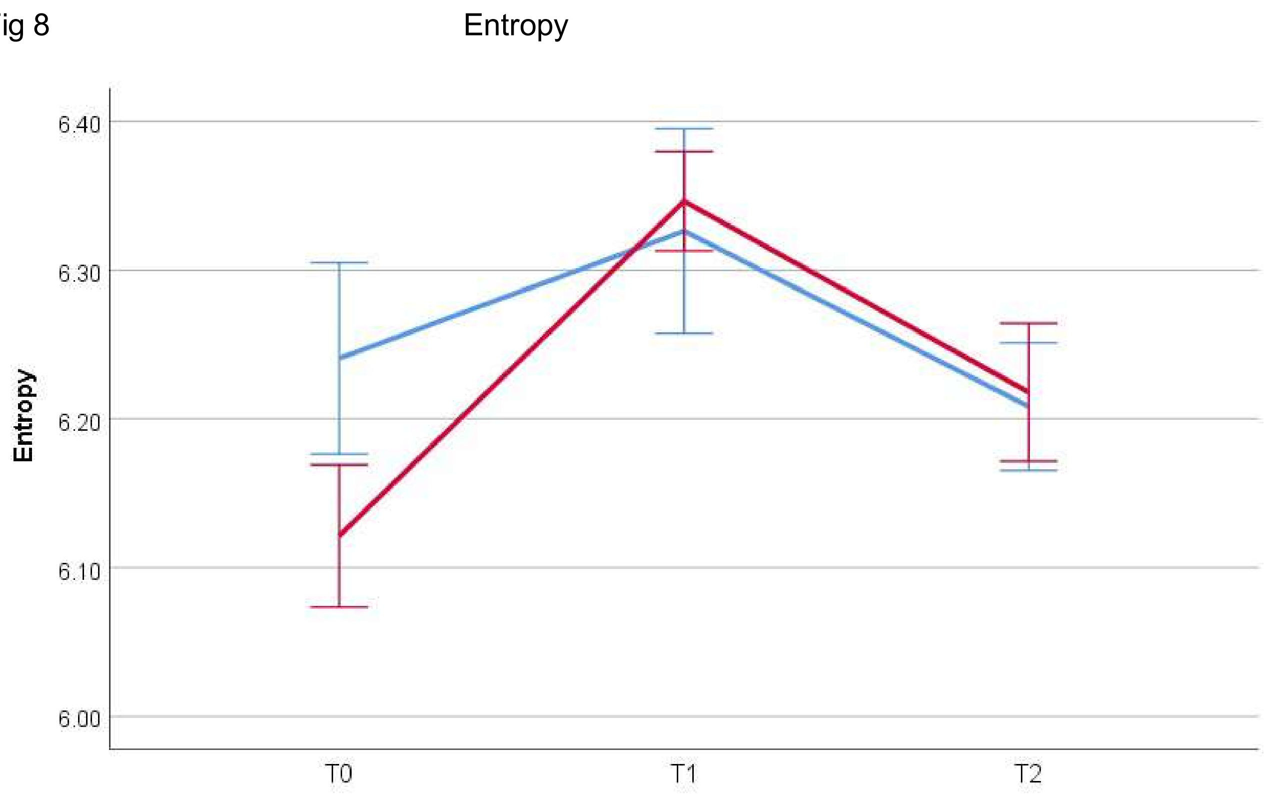
Entropy
Entropy
6.40
6.30
6.20
6.10
6.00
T0
T1
T2

Fig 9 VOH

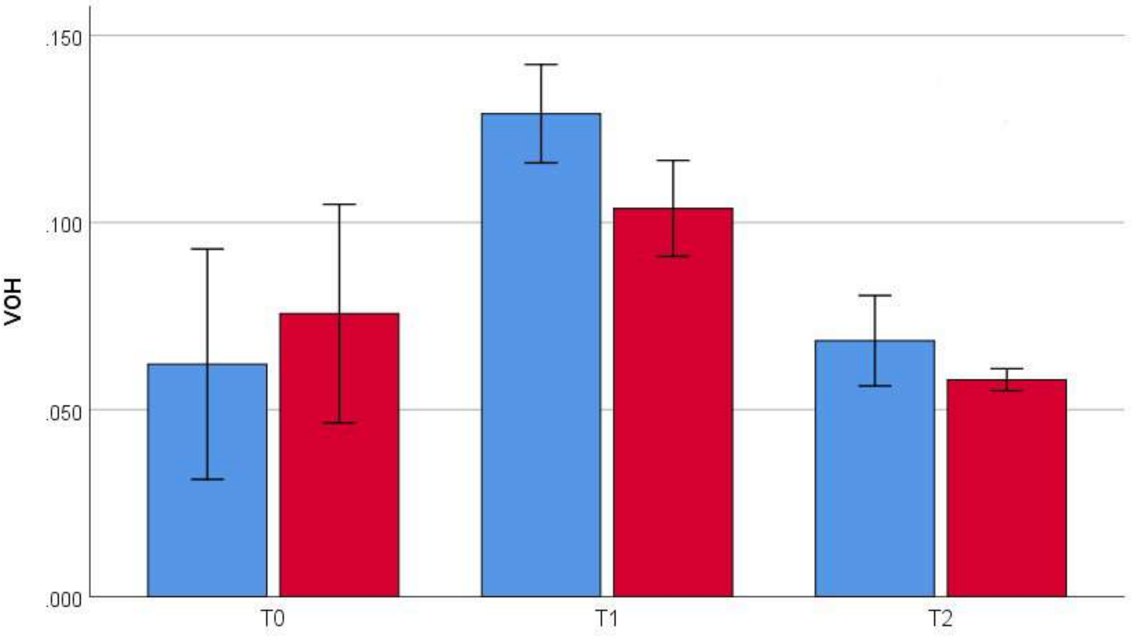

Fig 10 VOH

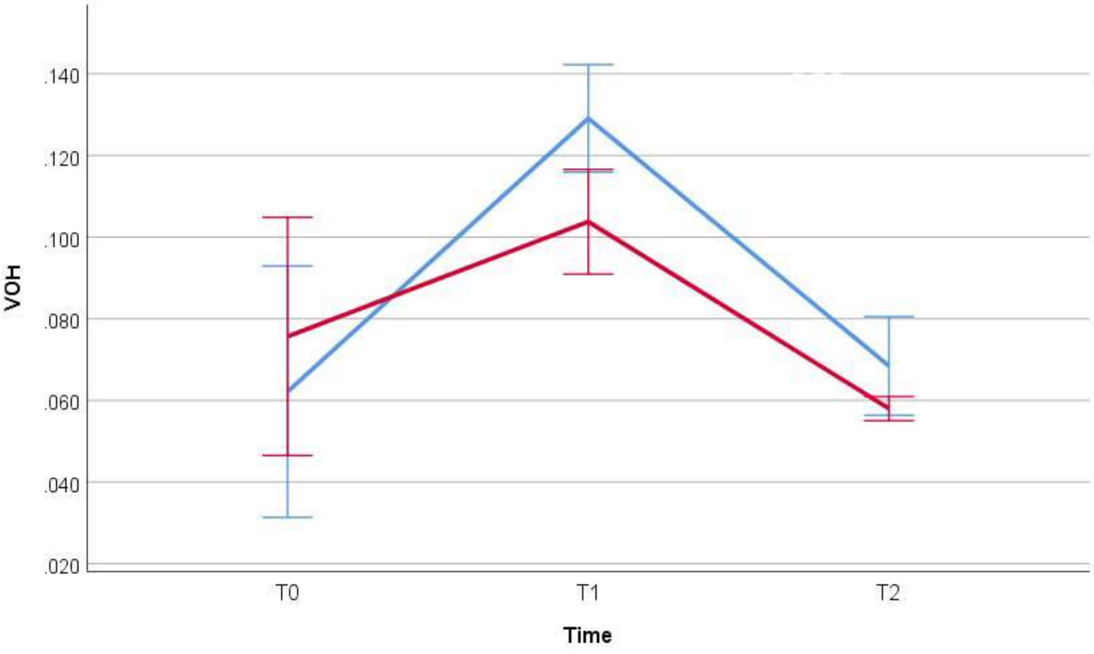

Supplement: S1 Appendix — (PDF) [file pone.0250936.s001.pdf]
