# Supplementary material for: The use of digital texture image analysis in determining the masticatory efficiency outcome
Source: PLoS One. 2021 May 6;16(5):e0250936. doi: 10.1371/journal.pone.0250936 (PMC8101913; doi:10.1371/journal.pone.0250936)
Supplement: Attachment — Submitted filename: Response to Reviewers.docx [file pone.0250936.s003.docx]

Dear Editor Dr Fabian Huettig,

Thank you for having considered our paper entitled: " Within diabetic subject evaluation of masticatory efficiency using digital image texture analysis"

We would sincerely like to thank you for your comments and suggestions that hopefully helped us to enhance the quality of the article. The manuscript is adjusted as you pointed and at the same time modified where we deemed it was necessary to strengthen the manuscript. Changes were highlighted in the text colored in yellow.

We thank you for considering this reviewed manuscript once again and hope we reassessed it properly for you to send it further into the procedure.

In the following section we also provided answers to each specific issue raised by your side.

a) *you state in the introduction that edentulism is a prevalent problem - please give references. Actually edentulism is decreasing across Europe – but of course not world wide.*

As you have pointed the referencse are added in the text, and the paragraph was rewritten.

b) *your number of patient (N=19) with DM is rather low for sophisticated statistical methods and p-value fishing. Thus, I invite you to better depict the graphical data with representation of the deviations by single dots of each measurement to allow a*

Our statistician has made changes as you requested.

Also we were aware of the small sample size at the beginning and addressed it in the Discussion section: “The limitations of this study may be in the small sample size, as well as in the shortcomings of chewing tests. However, evaluation of the literature dealing with similar topics revealed the optimal sample sizes. (34.Van der Bilt A, Speksnijder CM, De Liz Pocztaruk R, Abbink JH. Digital image processing versus visual assessment of chewed two-colour wax in mixing ability tests. Journal of Oral Rehabilitation.2012; 39: 11–17.

35.Miyaura K, Morita M, Matsuka Y, Yamaashita A, Watanabe T. Rehabilitation of biting abilities in patients with different types of dental prostheses. Journal of Oral Rehabilitation. 2000;27: 1073–1076.

36.Gunne H S, Wall A K. The effect of new complete dentures on mastication and dietary intake. Acta Odontologica Scandinavica. 1985;43: 257-268)

c) *more qualitative approach to your results and findings. This could include to track the performance (or measurements) of each individual from T0->T2 graphically.*

Our statistician has made changes as you requested.

d) *I am missing the STROBE checklist as well as data about the drop outs, exclusions and reasons for exclusion*.

Beginning of the Materials and method section was rewritten and modified accordingly in order to present inclusion and exclusion criteria more clearly.

e) *You assessed the Kapur score - please include the data to your findings and as highlighted in c) contrast the distributions of this score to the findings in your variables under evaluation.*

The paragraph was rewritten since you revealed the lack of clearness. The Kapur scoring was done during the selection of the participants in order to eliminate potential confounders that may influence the results like improper anatomy of the bearing tissues and inadequate clinical quality of the dentures.

f) *These measures may enable a better understanding of the results especially the divergence between VOH and contrast from T0->T2*

The values of VOH and contrast are not supposed to be correlated but both show similar trend of diminution proposing the fact that there is improvement of masticatory efficiency from T0-T2. Smaller values better color mixing and thorough mastication respectively.

decreasing

g) *please only enclose high resolution images in line art (all Figures in TIFF are depicted awfully - please check conversions or extraction paths*)

The Figures were corrected as you proposed.

h) *please help the reader to understand the variables more clearly. For me, the differences in variables under observation are quite small. How does dentate patients perform in this test? This is crucial because statistical significance has nothing to do with relevance.*

In Method and material section we included explanation of the variables in order to have deeper insight into the study results.

i) *To understand deviations please give the data of both cycles (I assume but you didnt mentioned that you used the mean of both chewings) and perform an intra-individual analysis about the reproducibility at each T. Therefore, I recommend to work rather with differences and deviations from the common mean than with extraorinary statistical methods.*

Part of the manuscript involving statistics analysis was rearranged. As a result of that new Tables 1 & 2 are introduced, and the results are better graphically presented in Figures 4 & 5.
