# Supplementary material for: The use of digital texture image analysis in determining the masticatory efficiency outcome
Source: PLoS One. 2021 May 6;16(5):e0250936. doi: 10.1371/journal.pone.0250936 (PMC8101913; doi:10.1371/journal.pone.0250936)
Supplement: Attachment — Submitted filename: Response to Reviewers.docx [file pone.0250936.s004.docx]

Response to the academic editor

*I'd like to invite you to adapt your manuscript towards the points raised by reviewer #1. I am following these points and stay with him to rather adjust your "story" and to reperform ex ante calculations and to*

*provide assumptions for your calculations in order to validate and present your method rather than the data itself (story lost).*

We accepted with great pleasure each point raised by yourself and the reviewer, and are enclosing the new version of the manuscript with changed „ story" and detailed focus on the method implemented. The introduction section is now completely rewritten, with different aims and issues raised. Further text is changed accordingly to meet the new approach in the manuscript. Also we have made changes to the title to better match the text itself.

Thanks to you we hope that our new version of the manuscript possess merit for the PLOS ONE journal.

Response to Reviewers

Reviewer #1: 1) The overall intention of this paper remains unclear due to the mix of all the paramenters: diabetic patients, full dentures and a new method to evaluate chewing efficiency.

*1a) the used technology was used first time according to the authors - is the method valid and robust? After having measured all the different data - uniformity, contrast, homogeneity, entropy - contrast turned out to be the best marker - but this is post hoc and was not predefined as hypothesis. The authors´conclusion is focused only on that point, diabetes and full dentures are completly ignored in the conclusion. The goal of the study was to see how their exposure to new CDs affects their masticatory performance as treatment outcome (line 72-73). It seems, that this focus was lost somehow.*

Thank you very much for your comment, because it forced us to thoroughly go through the text and make substantial corrections. Firstly, as you suggested we focused on the novel method and greatly rewrote the introduction with specific ephasize on the existing methods for evaluating masticatory efficiency. Further we added some desctirption regarding DTIA and its contemporary use in dental research so far. As you pointed, we introduced new aims with special focus on evaluating texture features in masticatory efficiency follow up, measuring chewing ability improvement and justifying the use of DTIA in masticatory efficiency evaluation.

*1b) it remains unclear, why diabetic patients have been selected - is there a difference to non-diabetic patients in chewing performance and/or adaptave capacity to new CDs? So, it is difficult to understand the clinical implications. A specific diabetic related altered learning period in diabetic patients cannot be identified in the conclusion. So, the lake of a control group is critical, especially in combination with the low number of participants.*

*As pointed in the Recommendations: Clear focus on the new mehtod or on DM in combination CD. Maybe two split into two separated papers?*

We rewrote our manuscript with completely different focus, abandoning diabetic patients and focusing only on thourough research about the texture analysis and methodical approach with the chewing gum testing. However, we felt that new wearers are representative group for our methodological approach since they as a new wearers certainly need more time to learn to cope with new dentures. Therefore, we were able to follow them in adequate period of time, providing our method to express itself in full.

2) *Statistics: there are no information about a repeated measurement design.*

Every participant chewed two chewing gum samples, according to the described procedure (line 159) in methodology section. And In Table 1 each parameter was presented with test-retest values.

3) *Age: the range 55 to 80 is wide, and in accordance to the literature (e.g. Peyron MA, Blanc O, Lund JP, et al. Influence of Age on adaptability of human mastication. JNeurophys 2004; 92;773-9 https://doi.org/10.1152/jn.01122.2003), age is an influencing factor. A subgroup analysis for different age groups (at least two) should be included. It is an important influencing factor, that the adaptive capacity is completely different in elderly compared to younger patients groups*.

We are completely aware of the fact you were pointing but age 55 was completely writing mistake, since we at the first place focused on older subjects our inclusion criterion was age over 65, which was written in the methodology section.

4) *Why the chewing test was performed on the preferred chewing side? Why not on both sides? Was preferred chewing side checked again at T1 and T2 - changes can be assumed in adequate and symmetric CD*.

The used methodology about mixing ability test with chewing double coloured chewing gum was adopted from the literature. 15.	Schimmel M, Christou P, Herrmann F, Mȕller F. A two-colour chewing gum test for masticatory efficiency: development of different assessment methods. Journal of Oral Rehabilitation. 2007; 34: 671–678. In order to completely create the real life situation we accepted the proposed the methodology.

Overall:

*The parameters diabetic patients, full dentures and a new method are not clearly separated and specified - so the interested clinician might expect an information regarding diabetes or full denture masticatory performance, and not a methodologic study on a new method.*

Thank you very much for your comments, because you opened my eyes and I have written the text in completely different manner. Sincerely hope that this new version of the mansucript will satisfy your expectations, because we really put a great effort on it. Of course the merit is on you, but hope we deserved it.

Recommandation:

Clear focus on the new mehtod or on DM in combination CD. Maybe two split into two separated papers?

This means that you should reflect the focus of your manuscript and rewrite it.

As the reviewer pointed out that your data are not suitable to cover findings in diabetic patients.

I am with the reviewer that it does not help do adjust the statistical methods to make your story work.

But, you did a thourogh research about the texture analysis and methodical approach with the chewing gum testing.

In hope this was helpful for you

I remain with best regards,

Fabian Huettig
