# Supplementary material for: The use of digital texture image analysis in determining the masticatory efficiency outcome
Source: PLoS One. 2021 May 6;16(5):e0250936. doi: 10.1371/journal.pone.0250936 (PMC8101913; doi:10.1371/journal.pone.0250936)
Supplement: Attachment — Submitted filename: Manuscript_R2-revision3.docx [file pone.0250936.s005.docx]

**The use of digital texture image analysis in determining the masticatory efficiency outcome**

Short title: Digital texture image analysis in masticatory efficiency evaluation

Aleksandra Milić Lemić^1*^^¶^, Katarina Rajković^2¶^, Katarina Radović^1&^, Rade Živković^1&^, Biljana Miličić^3¶^, Mirjana Perić^1&^

^1^ Clinic for Prosthetic Dentistry, Faculty of Dental Medicine, University of Belgrade, Belgrade, Serbia

^2^ College of Applied Studies of Technics and Technology, Kruševac, Serbia

^3^ Department for Informatics and Biostatistics Faculty of Dental Medicine, University of Belgrade, Belgrade, Serbia

*Corresponding author:

e-mail: [aleksandra.milic@stomf.bg.ac.rs](mailto:aleksandra.milic@stomf.bg.ac.rs) (AML)

ORCID ID: orcid.org/0000-0002-1549-7748

¶ These authors contributed equally to this work.

& These authors also contributed equally to this work

**Abstract**

The mixture level of gum samples consiting of two colors can be assessed visually, using the electronic colorimetric method, employing digital image processing techniques and specially designed software.

The study investigates the possibility of an alternative method called “digital texture image analyses” (DTIA) to assess improvement of masticatory efficiency in denture wearers. The objectives were i) to evaluate whether DTIA discriminate changes of the color mixing ability within a group over time; ii) to determine whether DTIA might be used to detect improvement in chewing ability iii) to select the most appropriate DTIA feature that sufficiently describe masticatory efficiency in CDs wearers;

The study was designed as an intra-individual evaluation of masticatory efficiency and was assessed in participants with new dentures in three follow-up times.

Statistically significant difference in masticatory efficiency was observed when comparing the measurements of gum samples at the three points in time, based on the Friedman’s test. Further analysis of the acquired results included the Wilcoxon test, a comparison between different measurement times. The examined gum samples, in all three time periods, showed statistically significant differences between the baseline (T0), after three months (T1) and after six months (T2). A set of four texture features were used in the current study. Uniformity, Contrast, Homogeneity and Entropy of obtained chewing-gum samples were corelated to the degree of gum comminution. Evaluation of the obtained results showed that Contrast and Homogeneity might be identified as being the best marker of gum degradation and reliable for observing the masticatory efficiency in denture wearers.

DTIA proved that it successfully recognises changes in functional parameters of masticatory efficiency in complete denture wearers.

**Introduction**

It is indisputable that tooth loss and edentulism have an impact on masticatory function and a consequent influence on the nutritional status [1,2,3]. Although, the prevalence of edentulism has fallen markedly in industrialised countries, [4,5] tooth loss is among the main conditions of interest when considering oral diseases among the elderly [6], who are already affected by other systemic diseases. Deterioration of masticatory function influences the efficiency of food absorption and nutritional status [7], limiting the food selection and dietary intake of fibres, magnesium or calcium [8,9], with potential effect on systemic health.

Edentulous people are likely to be treated with complete dentures (CDs) in order to rehabilitate their masticatory function to some extent. The ability to successfully wear a pair of CDs is, to an extent, a learned, skilled performance. Thus, it is expected that aged subjects first time CDs wearers may need time to cope with a new pair of dentures. After insertion of the complete dentures, the chewing ability improves as the patients get accustomed to their new dentures and the improved fit optimises retention and stability within the limits allowed by the anatomical and tissue conditions [10]. Chewing ability expressed as masticatory efficiency is a dynamic chewing function and objectively is evaluated by observing, measuring or quantifying food changes during mastication [11]. Various methods characterized as objective ones were used in masticatory efficiency assessment so far: Researchers evaluated changes in chewed natural foods [12], or synthetic food [13], as in sieving methods. The sieving method, which measures the comminution of the test materials, was considered the gold standard for assessing the masticatory efficiency especially multiple sieve method which yields better results than the single one [14]. A substitution of comminution tests is the ability to mix and knead a food bolus, simulated by usually two-coloured chewing gum [15, 16] or paraffin wax [17, 18], which was also widely used in masticatory efficiency evaluation [19]. The degree of mixing of the two colours was determined by optical methods [17, 19], by visual inspection [20] or by both [21]. Later, software was introduced for the image processing of the two-colour chewing gum mixing [22]. Although, colorimetric detectability of chewing-gum colour changes has proven to be a reliable method in clinical and academic masticatory efficiency assessment. These methods allow the gum colour changes but lack deeper insight into the changes of gum samples texture appearance. We hypnotise that using a “digital texture image analyses” (DTIA) might provide a better insight into the improvement of masticatory efficiency in denture wearers. DTIA is based on grey level co-occurrence matrix (GLCM) method where texture of an image corresponds to the spatial organisation of pixels in the image and the co-occurrence matrix describes the occurrence of grey level between two pixels separated in the image by a given distance [23]. There are up to 14 textural features of DTIA which may be selected to represent the textural characteristics of the image under investigation [23].

The DTIA method was used to evaluate the structure of osteoporotic bone based on series of images [24], follow the periapical bone healing [25] or differentiate soft tissue lesions [26]. Also, texture image analysis was used to characterise the impact of chewing on pasta particle size reduction [27] or food bolus characterisation [28]. Although, used sporadically in dental research, there are no publications about the application of DTIA in assessment of the digital images of chewed two-colour gums during the mixing ability test in masticatory efficiency evaluation.

Therefore, we investigated the applicability of DTIA in evaluating and measuring bolus-kneading capacity of a two-coloured chewing gum samples in complete denture wearers. The objectives were i) to evaluate whether DTIA discriminate changes of colour mixing ability within group in follow up time; ii) to determine whether DTIA might be used to detect improvement in chewing ability iii) to select the most appropriate DTIA feature that sufficiently describe masticatory efficiency in CDs wearers;

**Materials and method**

The study was designed as a prospective intra-individual cohort investigation of the masticatory efficiency in edentulous subjects with new CDs over 6 months. Following the requirements of the Declaration of Helsinki for ethical principles for medical research involving human subjects and according to the STROBE guidelines, prior to the very beginning of the investigation the study protocol was approved by the Ethics Committee of the School of Dental Medicine, University in Belgrade (No 35/14). All participants were given a detailed explanation of the purpose and process of the study, and gave their written informed consent. The investigation was conducted from October 2017 to July 2018 (including recruitment, clinical procedures, follow‐up after three and six months, and data collection).

**Inclusion and exclusion criteria**

Participants were recruited from a pool of patients who visited the Clinic of Prosthodontics, asking for a prosthodontic rehabilitation. They had undergone a clinical suitability examination and after	fabrication of upper and lower CDs and based on the predefined eligibility criteria a study group was formed.

Age over 65 for both sexes with controlled diabetes mellitus with oral hypoglycaemic medication and no additional systemic disease. Edentulous without previous experience in wearing CDs, fabricating a first pair of complete dentures. In order to eliminate potential confounders, another eligibility criterion was included such as normal volume and resilience of participants alveolar ridges providing good denture-bearing tissues [29, 30]. The last but not the least inclusion criterion was clinical quality of dentures. Clinical quality ratings of each patient’s dentures were assessed during the CDs insertion phase, regarding their stability and retention, as well as vertical dimension of occlusion and centric occlusion of the two dentures, respectively [31]. The methods for assessing the clinical quality of dentures and tissue support have been used according to literature data, and dentures were considered reliable for the study when the sum of scores indicated clinically good dentures according to Kapur [29]. Concurrently, confounding factors have been minimized since the influence of ill-fitting dentures, improper denture bearing tissues and other factors were eliminated, allowing that the cause (Diabetes mellitus) and effect (masticatory efficiency) may be distinguished.

Individuals who requested implant-supported appliances or demanded previous surgical interventions, verified allergies to any of the components of the CDs, subjects with signs of TMJ dysfunctions or neuromuscular disorders were excluded from the study. Participants who were unable to use the dentures continuously or who were unwilling to attend the follow-up appointments were excluded from the study, as well. Furthermore, the additional exclusion criteria were the sum score derived from the Kapur index for quality for denture-bearing tissues less than 14, and the sum of score for denture quality less than 6.

After the insertion of a new pair of CDs, verbal and written instructions about CDs insertion, removal, cleaning, and care were given to all participants.

**Test sample and chewing protocol**

Masticatory efficiency was assessed according to the described study protocol, by measuring the bolus-kneading capacity through the ability to mix a two-coloured chewing gum [32].

Samples for testing were prepared from Five Tape sugarless gum (The Wrigley Company Ltd, Plymouth, Devon, PL6 7PR, England) a tape of green layer and a tape of pink colour. Dimensions of chewing gum samples were adopted from literature [15,21]. Therefore, two pieces of gum tapes (length of 30 mm) were manually joined forming one sample with two different color layers, overall dimensions of 30mmx18mmx3mm. Although commercial chewing gums that have not been used before were implemented in the study, it was found that gum adhered to the specifications for aimed specimens, for use in the two-colour mixing ability test published prior [21].

**Clinical protocol and image assessment**

Upper and lower CDs were inserted and a sample of gum was placed onto the patient’s tongue with the pink side oriented towards the palate. Each subject was instructed to sit upright and to chew the sample of gum on the preferred chewing side for 20 cycles [15]. The chewing cycles were evaluated by the operator, who counted rhythmic open-close movement of the lower jaw in the anterior plane as one chewing stroke [33,34].

Every participant chewed two chewing gum samples, according to the described procedure; therefore, there were a total of two samples per subject for analysis. Between two consecutive chewing cycles an interval of 1 min was imposed in order to minimize the effect of fatigue. Immediately after the final chewing cycle, the samples were placed directly into a transparent plastic bag labelled with an identification code. Each specimen was compressed to a 1mm-thick piece using a custom-made polyvinyl chloride plate with a milled depression according to the described protocol [15]. Both sides of the piece were photographed with a Nikon D3200 digital camera using 18-55 mm lens and Macro filter 8x, (Nikon Corp. Japan) and saved in JPEG format as described in the literature [32].

**Digital texture image analysis (DTIA)**

Homogeneity, contrast, uniformity and entropy were set as independent variables of DTIA features, matching them with the level of masticatory performance. Each chewed specimen was analysed from both sides, so that an image of both sides of the gum was acquired as follows: The schematic presentation of the image processing procedure is shown in Fig 1. All images were converted from red, green, and blue (RGB) images (Fig 1 A) to grayscale (GS) images (Figure 1 B) applying….(Software). Grey Level Co-Occurrence Matrix (GLCM) algorithms were applied to obtain texture features.

**Fig 1. The schematic presentation of the image processing procedure.** The red, green, and blue images (RGB) and the grayscale (GS) images.

The entire DTIA methodology was carried out using the Image J program, v 1.34s (National Institutes Health, Bethesda, MD, USA). The GLCM is the second-order statistic algorithm that compares two neighbouring pixels at a time and compiles the frequency at which different grey-levels can be found within a restricted area [35,36]. As mentioned above four digital texture variables were measured in this study: uniformity, contrast, homogeneity and entropy.

The uniformity represents the texture uniformity of the image and it is the opposite feature of entropy. It measures the number of repeated pairs, which is expected to be high if the occurrence of repeated pixel pairs is high [37]. The function was called from …. Applying the adjustment ….. .

The contrast is a measure of local variations of grey level values of pixels in the images [36]. Homogeneity measures the local homogeneity of a pixel pair. The homogeneity is expected to be large if the grey levels of each pixel are similar [37]. Entropy measures the disorder, or randomness of images, and can be used to characterize the image texture. It is an indicator of the complexity within an image, so, the more complex the images, the higher the entropy values [36,38,39]. A smaller difference in the levels of the grey scale of the pixels in the image corresponds to lower values of contrast and entropy, presenting better colour mixing and consequently the better the masticatory performance. Also, smaller difference in the levels of the gray scale of the pixels in the image are in relation to higher values of uniformity and homogenity, meaning better colour mixing and consequently efficient masticatory performance. The digital texture features used were also described by Tournier et al. [23].

**Colorimetric analysis**

Colorimetry (analysis of variance of the hue) was performed using the freeware ViewGum© software (dHAL Software, Greece, www.dhal.com), as described previously [22].

Obtained images of specimens were imported into the custom ViewGum software. As seen in Fig 1A after segmentation the images were transformed from RGB values (red, green, blue) to the HSI colour space (hue, saturation, intensity) and further, the hue value for each pixel in the semi automatically segmented images was calculated [22]. The variance of hue (VOH) was determined as the measure of level of colour mixture [21], ranging from 0 to 1, which represents fusing the two colours into a single colour where the lower VOH value, the corresponds to better colour mixing and consequently the better the masticatory performance.

**Statistical analysis**

Statistical analyses were performed using SPSS software (Version 26, COMPANY; Chicago,IL, USA). All obtained values were numerical. Descriptive data were expressed as mean, standard deviation, median and the interquartile range. Numeric data were tested for normal distribution using the Koglomorov-Smirnov test. All our data were non-parametric, hence the Fridman test and Wilcoxon test were used to analyse differences between the data at different follow‐up times and baseline. Mann Whitney test was used for intra-individual comparation. Differences were considered significant when p-value was <0.05.

**Results**

**Participants**

After all the study included 19 participants with the mean age 68 ± 7.6 (range 65 to 80 years), and all of them completed the two follow-up visits. All obtained chewing samples (N=114) were suitable for further evaluation. The study group gum samples were obtained at three different time periods and three subgroups of samples were formed: at the baseline (T_0_), after 3 months (T_1_) and after 6 months (T_2_), Representative samples of chewing gum mixing ability images for T_0_, T_1_, and T_2_ are shown in Figure 2 as A, B and C.

**Fig 2. Representative samples of chewing gum mixing ability images for T0, T1, and T2**

Each patient chewed two samples of chewing gum, so there were 38 chewed gum samples per subgroup with total of 114 chewed samples. Since images were taken from both sides there were a total of 228 images that were analysed using DTIA and VOH. A flow diagram of participants and total gum samples is presented in Fig 3.

**Fig 3. Flow diagram of participants and total gum samples implemented in the study.**

The main variables of investigation are presented in Table1. Based on intra-group comparation between chewed samples of both cycles significance was observed in between Uniformity group at the baseline and after three months, for Entropy at the baseline and after three months for VOH. Furthermore, from the values presented in Table 1, one may say that the values of the analysed variables, Uniformity, Contrast, Homogeneity and Entropy, of gum samples have changed in participants during the observation period, from the moment of CDs delivery (T0), after three months (T1) and after six months (T2). Also, a statistically significant change of VOH in the tested gum samples was noticed for all three follow-up times, which can also be seen in Table 1.

**Table 1. Parameter values in study group at the baseline, after three and after six months**

| ***Variables*** | ***Baseline (T0)*** | ***After three months (T1)*** | ***After six month (T2)*** | ***^#^Significance*** |
| --- | --- | --- | --- | --- |
| ***Uniformity(1)*** | 0,0033 (0,000575)  *0,0030 (0,000)* | 0,0028 (0,0008)  *0,0030 (0,001)* | 0,0031 (0,00024)  *0,0030 (0,0000)* | ^a^p=0,010* |
| ***Uniformity(2)*** | 0,0034 (0,000575)  *0,0033 (0,000)* | 0,0027 (0,0003)  *0,0028 (0,00022)* | 0,0030 (0,0008)  *0,0030 (0,001)* | ^a^p=0,001* |
| ***^&^*** ***Significance*** | ^b^p=0,029* | ^b^p=0,000* | ^b^p=0,318 |  |
| ***Contrast (1)*** | 2,870 (0,641)  *2,645 (0,719)* | 2,365 (0,414)  *2,273 (0,262)* | 2,098 (0,356)  *2,043 (0,511)* | ^a^p=0,000* |
| ***Contrast (2)*** | 2,676 (0,471)  *2,773 (0, 196)* | 2,337 (0,116)  *2,351 (0,108)* | 2,168 (0,288)  *2,133 (0,272)* | ^a^p=0,000* |
| ***^&^*** ***Significance*** | ^b^p=0,974 | ^b^p=0,142 | ^b^p=0,508 |  |
| ***Homogenity(1)*** | 0,539 (0,024)  *0,542 (0,030)* | 0,560 (0,019)  *0,566 (0,022)* | 0,576 (0,025)  *0,581 (0,038)* | ^a^p=0,000* |
| ***Homogenity(2)*** | 0,547 (0,010)  *0,543 (0,043)* | 0,564 (0,008)  *0,562 (0,006)* | 0,569 (0,020)  *0,572 (0,018)* | ^a^p=0,002* |
| ***^&^*** ***Significance*** | ^b^p=0,420 | ^b^p=0,936 | ^b^p=0,497 |  |
| ***Entropy (1)*** | 6,24 (0,13)  *6,26 (0,173)* | 6,326 (0,138)  *6,31 (0,124)* | 6,208 (0,086)  *6,205 (0,134)* | ^a^p=0,000* |
| ***Entropy (2)*** | 6,12 (0,096)  *6,07 (0,067)* | 6,346 (0,067)  *6,33 (0,089)* | 6,222 (0,086)  *6,215 (0, 047)* | ^a^p=0,000* |
| ***^&^*** ***Significance*** | ^b^p=0,006* | ^b^p=0,323 | ^b^p=0,386 |  |
| ***VOH (1)*** | 0,062 (0,062)  *0,050 (0,095)* | 0,129 (0,026)  *0,127 (0,037)* | 0,068 (0,024)  *0,066 (0,034)* | ^a^p=0,000* |
| ***VOH (2)*** | 0,076 (0,059)  *0,068 (0,090)* | 0,104 (0,026)  *0,116 (0,052)* | 0,058 (0,006)  *0,062 (0,011)* | ^a^p=0,005* |
| ***^#^*** ***Significance*** | ^b^p=0,235 | ^b^p=0,020* | ^b^p=0,299 |  |

Mean (SD); Med (IQR); *statistically significance; #comparation between follow-up periods; &intergroup comparation; ^a^Fridmanov test; ^b^Mann Whitney test

Similarity between test-retest samples is observed in Contrast and Homogeneity values for all three follow-up times as seen in Table 1, while concurrence in test-retest samples is found for Entropy in T_1_ and T_2_ intervals. Values for Uniformity successive measurements of one subject were significantly different at the beginning and three months after, and for VOH after three months.

Further analysis of the acquired results included the Wilcoxon test, a comparison between different measurement times, as shown in Table 2. Furthermore, statistically significant changes in values are seen, especially when comparing the parameters Uniformity, Contrast and Homogeneity at the baseline (T0), with regards to the values after three months (T1) and after six months (T2). More graphically presentations of the variable values are seen in Fig 1-10 in S1 Appendix. When observing the VOH values of the examined samples in all three time periods, statistically significant differences between the baseline (T0), after three months (T1) and after six months (T2) were noticed, as well as between the values of T1 and T2. (Fig 11 &12 in S1 Appendix)

**Table 2 Values for intergroup comparation**

| ***Variables*** | ***Follow-up time*** | ***T0*** | ***T1*** |
| --- | --- | --- | --- |
| ***Uniformity (1)*** | T1 | p=0.052 |  |
|  | T2 | p=0.248 | p=0.102 |
| ***Uniformity (2)*** | T1 | p=0.002* |  |
|  | T2 | p=0.296 | p=0.032* |
| ***Contrast (1)*** | T1 | p=0.001* |  |
|  | T2 | p=0.000* | p=0.001* |
| ***Contrast (2)*** | T1 | p=0.016* |  |
|  | T2 | p=0.000* | p=0.030* |
| ***Homogenity (1)*** | T1 | p=0.002* |  |
|  | T2 | p=0.010* | p=0.004* |
| ***Homogenity (2)*** | T1 | p=0.000* |  |
|  | T2 | p=0.000* | p=0.443 |
| ***Entropy (1)*** | T1 | p=0.048* |  |
|  | T2 | p=0.459 | p=0.002* |
| ***Entropy (2)*** | T1 | p=0.000* |  |
|  | T2 | p=0.000* | p=0.000* |
| ***VOH (1)*** | T1 | p=0.001* |  |
|  | T2 | p=0.679 | p=0.000* |
| ***VOH (2)*** | T1 | p=0.048* |  |
|  | T2 | p=0.500 | p=0.000* |

*statistical significance; Wilcoxon test

**Discussion**

**Summary of the results**

Implementing DTIA enabled a thorough masticatory efficiency analysis, with discrimination of masticatory efficiency progression within the edentulous subjects during adaptation time. Obtained results give insight into how fast the process of adaptation is going in edentulous patients first time CDs wearers in relation to masticatory efficiency. The implemented method of DTIA is new and it has been supplemented with the already published and accepted colour mixture VOH analysis [15,21,22,34], although they analyse different aspect of chewed gum samples both analyses proved masticatory efficiency, discriminative to analysed study group during the observed time, and DTIA was found consistent with the colorimetric method which was considered as gold standard. Also, the DTIA possess good reliability for two texture features, since there were similarity between the test-retest samples from the same subject for Contrast and Homogeneity. Therefore, Contrast and Homogeneity might be identified as textural feature marker that could quantify chewing ability.

**Strength and weakness of the study**

Although some authors designate the sieving test as the most reliable method of mastication analysis [32,40], the chewing gum test was reported as a reliable method for quantifying masticatory performance [30], especially in CD wearers [16,19,32], with compromised mastication, as was the case in the conducted study. This type of test has many advantages: reduced possibility of swallowing food parts and, consequently, loss of analytical material, as well as the fact that the elasticity of the material allows the use of maximum available mastication capacity. Also, the mixing-ability tests for masticatory efficiency are less dependent on the flow rate of saliva, which is of particular importance in older people who often suffer from dry mouth symptom [40]. The duration of 20-cycle chewing was implemented in the study according to recommendations in literature as this seems to sufficiently discriminate between the ‘physiological’ chewing efficiency of a young, healthy and fully dentate person and the edentulous elderly individual with a substantial masticatory handicap [15]. The analysis of the inter and intra-individual reproducibility suggested that, for the application of the test, a fixed number of 20 chewing cycles should be applied. Also, it is reported that, when groups of participants are to be correlated, it is better to analyse 20 strokes, since it is not known after how many strokes the comparation curves keep diverging [41]. This number of chewing cycles was found useful, whereas high repeatability allows for follow-up evaluations, for example during a dental treatment or neuromuscular rehabilitation programs [21].

It is reported that a significant increase in masticatory efficiency can be obtained about 6 weeks after insertion of new complete dentures, while some authors stated that biting abilities improved 2 months after insertion of the new prostheses [42, 43]. Nevertheless, we performed the first follow up after three months. The idea was to give more time for new wearers to get used to dentures and perform a more realistic mixing ability test. Later, masticatory function test was performed 6 months after fabrication of good-quality complete dentures, as it was reported that during that period new muscle memory patterns for mastication would have been established [44].

Edentulous patients with adequate size of the residual alveolar ridge have been included in this study, in order to avoid impact of residual ridge resorption on masticatory function. Although, it was recently indicated that mandibular bone atrophy does not interfere directly with the masticatory function of edentulous patients [45].

The limitations of this study may be in the small sample size, as well as in the shortcomings of chewing tests. However, evaluation of the literature dealing with similar topics revealed similar sample sizes [41,42,43]. One drawback of the present study is the missing control group in the form of dentate subject. However, findings for the dentate are well documented elsewhere but data about edentulous subjects are lacking. The main shortcoming of the study may be the use of commercial chewing gum in the analysis of masticatory efficiency and not gum specially designed for this purpose. However, commercial chewing gums were used elsewhere in literature as specimens for chewing gum mixing ability test [21,46]. Also, when testing using this method in CDs wearers, one must take into account the possibility of the onset of irritation and pain caused by wearing dentures during the adaptation period, which may influence the masticatory efficiency of these patients. Hence, transitory problems such as discomfort, sore spots, and injured mucosa were taken care of at the very beginning, providing minimum impact on the conducted investigation.

**Interpretation of the results in the context of applied methodology**

Analysis of literature showed that for the analysis of the mixture level between the two colours of the gum, samples might be assessed both visually and by the electronic colorimetric assessment [32], or by using digital image processing techniques [15] and specially designed software [22]. Evaluation of the obtained results in mentioned procedures is more or less based on measurement of fusing the original colours into a new colour, with different intermediate intensities.

In this study, the chewing gum samples presented different visual aspects on RGB images (Fig. 2) which changed over the chewing periods from T_0_ to T_2_. However, images showed that the texture of chewing gum changed over the chewing sequences (Fig 2). After different chewing cycles, the chewing gum had variations in grey levels on images (Fig 1). As opposed to previous research we implemented DTIA that gave insight not only into the colour change, but to the texture of the digital gum image. The texture refers to a visual feature which is homogenous, but the homogeneity does not come from one colour. The texture features used in the study are variables such as uniformity, contrast, homogeneity, and entropy of obtained chewing gum samples pictures, which are in direct relation to comminution of the gum, and their values for each participant are presented in Figure 4. The uniformity refers to the existence of irregularities within the surface color of the specimen image and there will be fewer of them as the sample is “better” blended / chewed. The contrast measures the difference in the colour of the specimen, which means that it is reduced in well chewed samples, where the colours are well mixed. The homogeneity, in the context of measuring chewing efficiency, shows that a mixture of two colours was created, meaning the sample has been mixed and the colours merged into one. Entropy is a measure of image system regulation of the specimen. The smaller it is the sample is more arranged, or rather - better chewed.

**Fig 4. DTIA variables values for each patient during the observation period (at the baseline, after three months, after six months)**

Uniformity (A); Contrast (B) Homogeneity (C); Entropy (D).

Uniformity values, are the lowest at the first follow up as shown in the Fig 4A, showing the chewiness of the sample after the third month. Suggesting that the participants at the baseline faced with chewing gum samples between CDs masticated vigorously, thus affirming initial uniformity inside the sample. Lately, after a certain period of use (T_2_), when analysed again, their mastication was more coordinated, not demanding vigorous mastication. These findings in future research should be confirmed with parallel evaluation of masticatory force values.

Evaluation of the obtained results shows that the easiest method of observing the masticatory efficiency in edentulous subjects is by analysing Contrast and Homogeneity. Contrast, as a measure of chewed samples, shows a decrease during the observation period, as seen in Fig 4 B. This means that the contrast in the tested samples is reduced, due to a higher masticatory efficiency and more complete chewing. The values for Contrast were highest at the baseline, confirming that mixing ability is scarce, with distinguished colours in the samples and great contrast between colours. Contrast among all tested texture features showed compliance between test-retest samples in all three follow ups. The same is found for Homogeneity feature, which possess good reliability since there is also similarity between test retest samples from the same subject. Tournier et al. [23] arrived at the same conclusion which showed that contrast could be used successfully to investigate food bolus formation during chewing different breads.

Homogeneity parameter values are increasing from T_0_ toward T_2_ with statistical significance (Table 1), showing a gradual improvement of masticatory efficiency. The graph in Fig 4C shows an increase of masticatory efficiency in terms of homogeneity after three months and after six months, which indicates that there is an increase of masticatory efficiency at the beginning with slight during the course of observing the edentulous subjects.

Entropy values are shown on graph in Fig 4D. Entropy is in correlation with Uniformity and confirms the claims that the participants improve their masticatory efficiency after three months and that, by the sixth month, there are only improvements in the fineness of chewing.

The VHO values were lowest for the edentulous participants after three months, which is in correlation with findings that adequate chewing leads to well-mixed colours and a low VOH [21]. In addition, as seen in Fig 5, the highest VOH results were found in poorly mixed colours through deficient chewing, whereas in this study it was found at the beginning of the period of adaptation.

**Fig 5. VOH Values of Colorimetric analysis for each patient during the observation period (at the baseline, after three months, after six months)**

**Clinical implications**

Improving abilities to coordinate new dentures by tongue and muscle activity of the masticatory function are shown in relation to the mixing ability of two-coloured chewing gum which can be detected by variable values of the DTIA. The results of the DTIA were in support of the mentioned expectations, where analysed texture features confirmed a gradual improvement of masticatory efficiency in the study group through the evaluation period. The broad set of texture features used in the current study enabled refinement of masticatory efficiency evaluation. This suggests that DTIA and Colorimetric analysis, methods that incorporate different principles can be used as a control of one another for the chewing gum test of masticatory efficiency in clinical settings.

**Conclusion**

Accepting the discussed weakness of the study, it may be presumed that the DTIA can be regarded as a reliable tool to assess changes in the masticatory efficiency through mixing behavoir of two-coloured chewing gum samples. A hypothised increasement of masticatory efficiency was observed by DTIA which thereby seems to be sensitive enough to detect improvement in chewing ability. This is most predictable by contrast*,* and homogeneitychanges, but not uniformity*.* Further studies shall clarify the reproducibility and threshold of values to better interpret the data from DTIA alone as well as in hand with VHO.

**References**

1. Sheiham A, Steele JG, Marcenes W, Lowe C, Finch S, Bates C J, et al. The relationship among dental status, nutrient intake, and nutritional status in older people. Journal of Dental Research. 2001; 80: 408–413.
2. Hutton B, Feine J S, Moais J. Is there an association between edentulism and nutritional state? Journal of Canadian Dental Association. 2002; 8: 82–87.
3. Morais J A, Heydecke G, Pawliuk J, Lund JP, Feine J S. The effects of mandibular two-implant overdentures on nutrition in elderly edentulous individuals. Journal of Dental Research. 2003; 82: 53–58.
4. Sanders AE, Slade GD, Carter KD, Stewart JF. Trends in prevalence of complete tooth loss among Australians, 1979-2002. Aust N Z J Public Health 2004; 28: 549–54.
5. Sussex PV. Edentulism from a New Zealand perspective – a review of the literature. N Z Dent J. 2008; 104: 84–96.
6. Petersen PE, Yamamoto T.Improving the oral health of older people: the approach of the WHO Global Oral Health Programme. Community Dent Oral Epidemiol. 2005; 33: 81–92.
7. Ranawana V, Clegg M E, Shafat A, Henry CJ. Postmastication digestion factors influence glycemic variability in humans. Nutrition Research. 2011; 31: 452-459.
8. Bates J F, Stafford GD, Harrison A. Masticatory function‐a review of the literature. (III) Masticatory performance and efficiency. Journal of Oral Rehabilitation.1976; 3: 57– 67.
9. Manly RS, Braley L. Masticatory performance and efficiency. Journal of Dental Research. 1950; 29: 448– 462.
10. Leles CR, Oliveira TMC, Araújo SC, Nogueira TE, Schimmel M. Individual factors associated with masticatory performance of complete denture wearers: A cross‐sectional study. Journal of Oral Rehabilitation. 2019; 00: 1–9.
11. Jeong SH, Kang SM, Ryu JH, Kwon HK, Kim BI. Subjective food intake ability in relation to the Mixing Ability Index in Korean adults. Journal of Oral Rehabilitation. 2010; 37: 242–247.
12. Kapur KK, Soman SD. Masticatory performance and efficiency in denture wearers. 1964. Journal of Prosthetic Dentistry. 2006; 95: 407–411.
13. Ohara A, Tsukiyama Y, Ogawa T, Koyano K. A simplified sieve method for determining masticatory performance using hydrocolloid material. Journal of Oral Rehabilitation. 2003; 30: 927–935.
14. van der Bilt A, Fontijn-Tekamp FA. Comparison of single and multiple sieve methods for the determination of masticatory performance. Journal of Dental Research. 2002; 81: A–455.
15. Schimmel M, Christou P, Herrmann F, Mȕller F. A two-colour chewing gum test for masticatory efficiency: development of different assessment methods. Journal of Oral Rehabilitation. 2007; 34: 671–678.
16. Van der Bilt A, Mojet J, Tekamp FA, Abbink J H. Comparing masticatory performance and mixing ability. Journal of Oral Rehabilatition. 2010; 37: 79–84.
17. Sato H, Fueki K, Sueda S, Sato S, Shiozaki T, Kato M et al. A new and simple method for evaluating masticatory function using newly developed artificial test food. Journal of Oral Rehabilatition. 2003; 30: 68–73.
18. Salleh NM, Fueki K, Garrett NR, Ohyama T. Objective and subjective hardness of a test item used for evaluating food mixing ability. Journal of Oral Rehabilatition. 2007; 34: 174–183.
19. Speksnijder CM, Abbink JH, van der Glas HW, Janssen NG, van der Bilt A. Mixing ability test compared to a comminution test in persons with normal and compromised masticatory performance. European Journal of Oral Science. 2009; 117: 580–586.
20. Liedberg B, O¨ wall B. Oral bolus kneading and shaping measured with chewing gum. Dysphagia. 1995; 10: 101–106.
21. Schimmel M, Christou P, Miyazaki H, Halazonetis D, Herrmann F, Mȕller F. A novel colourimetric technique to assess chewing function using two-coloured specimens: Validation and application. Journal of Dentistry. 2015; 43: 955 – 964.
22. Halazonetis DJ, Schimmel M, Antonarakis GS, Christou P. Novel software for quantitative evaluation and graphical representation of masticatory efficiency. Journal of Oral Rehabilation. 2013; 40: 329-335.
23. Tournier C, Grass M, Zope D, Salles C, Bertrand D. Characterization of bread breakdown during mastication by image texture analysis. Journal of Food Engineering. 2012; 113: 615–622.
24. Kavitha, MS, An SY, An CH, Huh KH, Yi WJ, Heo MS, Lee SS, Choi SC. Texture analysis of mandibular cortical bone on digital dental panoramic radiographs for the diagnosis of osteoporosis in Korean women. Oral Surgery Oral Medicine Oral Pathology Oral Radiology. 2015; 119: 346–356.
25. Borowska, M.; Szarmach, J.; Oczeretko, E. Fractal texture analysis of the healing process after bone loss. Computerized Medical Imaging and Graphics. 2015; 46: 191–196.
26. Jurczyszyn K, Kozakiewicz M. Differential diagnosis of leukoplakia versus lichen planus of the oral mucosa based on digital texture analysis in intraoral photography. Advances in Clinical and Experimental Medicine. 2019; 28(11): 1469–1476.
27. Hoebler C, Karinthi A, Devaux, MF, Guillon F, Gallant DJ, Bouchet B, Melegari C, Barry JL. Physical and chemical transformations of cereal food during oral digestion in human subjects. British Journal of Nutrition. 1998; 80(5): 429–436.
28. Arvisenet G, Billy L, Poinot P, Vigneau E, Bertrand D, Prost C, Effect of apple particle state on the release of volatile compounds in a new artificial mouth device. Journal of Agricultural and Food Chemistry. 2008; 56 (9): 3245–3253.
29. Kapur KK. A clinical evaluation of denture adhesives. Journal of Prosthetic Dentistry. 1971; 28: 550-558.
30. Bradbury J, Thomason JM, Jepson NJA, Walls AWG, Mulvaney CE, Allen PF, et al. Perceived chewing ability and intake of fruit and vegetables. Journal of Dental Research. 2008; 87: 720-725.
31. Kapur K K, Garrett NR, Hamada MO, Roumanas ED, Freymiller E, Han T, et al. Randomized clinical trial comparing the efficiency of mandibular implant-supported overdentures and conventional dentures in diabetic patients. Part III: comparisons of patient satisfaction. Journal of Prosthetic Dentistry. 1999; 82: 416-427.
32. Silva LC, Nogueira TE, Rios LF, Schimmel M, Leles C R. Reliability of a two-colour chewing gum test to assess masticatory performance in complete denture wearers. Journal of Oral Rehabilitation. 2018; 45: 301–307.
33. Piccinato-Pirola M, Meli-Filho F, Trawitzki L. Chewing time and chewing strokes in different dentoafacila deformities J Soc Bars Fonoaudiol. 2012; 24(2): 130-133.
34. Buser R, Ziltener V, Samietz S, Fontolliet M. Nef T, Schimmel M. Validation of a purpose-built chewing gum and smartphone application to evaluate chewing efficiency. Journal of Oral Rehabilitation. 2018; 45: 845–853.
35. Gosselin R, Dushesne C, Rodrique D. On the characterization of polymer powders mixing dynamics by texture analysis. Powder Technology. 2008; 183: 77-88.
36. Arzate-Vázquez I, Chanona-Pérez JJ, Calderón-Domínguez G, Terres-Rojas E., Garibay-Febles V, Martínez-Rivas A, et al. Microstructural characterization of chitosan and alginate films by microscopy techniques and texture image analysis. Carbohydrate Polymers. 2012; 87: 289-299.
37. Wang H, Guo XH, Jia ZW, Li HK, Liang ZG, Li KC, et al. Multilevel binomial logistic prediction model for malignant pulmonary nodules based on texture features of CT image. European Journal of Radiology. 2010; 74: 124-129.
38. Fernández L, Castillero C, Aguilera JM. An application of image analysis to dehydration of apple discs. Journal of Food Engineering. 2005; 67: 185-193.
39. Mendoza F, Dejmek P, Aguilera JM. Colour and image texture analysis in classification of commercial potato chips. Food Research International. 2007; 40: 1146-1154.
40. Anastassiadou V, Heath MR. The development of a simple objective test of mastication suitable for older people, using chewing gums. Gerodontology. 2001; 18: 79–86.
41. Van der Bilt A, Speksnijder CM, De Liz Pocztaruk R, Abbink JH. Digital image processing versus visual assessment of chewed two-colour wax in mixing ability tests. Journal of Oral Rehabilitation. 2012; 39: 11–17.
42. Miyaura K, Morita M, Matsuka Y, Yamaashita A, Watanabe T. Rehabilitation of biting abilities in patients with different types of dental prostheses. Journal of Oral Rehabilitation. 2000; 27: 1073–1076.
43. Gunne H S, Wall AK. The effect of new complete dentures on mastication and dietary intake. Acta Odontologica Scandinavica. 1985; 43: 257-268.
44. Singhal S, Chand P, Singh BP, Singh SV, Rao J, Shankar R, et al. The effect of osteoporosis on residual ridge resorption and masticatory performance in denture wearers. Gerodontology. 2012; 29 (2012) e1059.e1066.
45. Marcello-Machado RM, Machado BA, Nascimento GG, de Rezende Pinto L, Del Bel Cury AA, Faot F. Masticatory function parameters in patients with varying degree of mandibular bone resorption. Journal of Prosthodontic Research. 2017;61: 315-323.
46. Weijenberg RAF, Scherder EJA, Visscher C M, Gorissen T, Yoshida E, Lobbezoo F. Two-colour chewing gum mixing ability: digitalisation and spatial heterogeneity analysis. Journal of Oral Rehabilitation. 2013; 40: 737-743.
47. Prinz JF. Quantitative evaluation of the effect of bolus size and number of chewing strokes on the intro-oral mixing of a two-colour chewing gum. Journal of Oral Rehabilitation. 1999; 26: 243–247.
48. Suzuki H, Fukushima M, Okamoto S, Takahashi O, Shimbo T, Kurose T, et al. Effects of thorough mastication on postprandial plasma glucose concentrations in nonobese Japanese subjects. Metabolism. 2005; 54: 1593–1599.
