# Supplementary material for: The use of digital texture image analysis in determining the masticatory efficiency outcome
Source: PLoS One. 2021 May 6;16(5):e0250936. doi: 10.1371/journal.pone.0250936 (PMC8101913; doi:10.1371/journal.pone.0250936)
Supplement: Attachment — Submitted filename: Response to Reviewers .doc [file pone.0250936.s006.doc]

Dear Reviewers:

Thank you for the opportunity to revise our manuscript, "The use of Digital texture image analysis in determining the masticatory efficiency outcome" (PONE-D-20-18199R2)

We appreciate the time and effort that the reviewers dedicated to providing valuable feedback on our manuscript. We also appreciate the careful review and constructive suggestions. We have been able to incorporate changes to reflect most of the suggestions made by the reviewers. It is our belief that the manuscript is substantially improved after making the suggested edits.

Following this letter are our point-by-point responses to the reviewers’ concerns in italics, including how the text was modified. The changes made in the manuscript are highlighted in yellow.

Reviewer #1:

Now the revised manuscript has a clear red line and is therefore adequately understandable.

*Thank you very much. Hope that the manuscript is even better after these modifications.*

Recommended adaptations:

a) Table 1: several Spaces missing in column Variables

*Changed*

b) Table 2: Spelling mistake: Entropy

*Changed*

c) Table 1 and Table 2: the * to highlight significance can be omitted (to relieve the content of the tables). The p-value is mentioned in the text (line 220 and 221)

*Thank you for this, as you recommended we omitted * in the Tables.*

d) Table 1 and Table 2: p<0,001 instead of p=0,000 (I guess that the probability of error is not really zero)

*Changed*

e) Table 1 and Table 2: the meaning of (1) and (2) remains unclear, although it seems to be essential for the understanding of the statistical analysis. (1) = DTIA?; (2) = VOH?. Recommendation: no coding, but insertion of the abbreviations.

*Thank you for your suggestion. We have inserted the meaning of (1) and (2) into the Tables and changed them accordingly. Didn`t quite understand what you meant by "no coding, but insertion of the abbreviations"*

f) Table 1 and Table 2: the number should be explained in the text - or I haven´t found it.

*We mentioned the table number in the text.*

g) The sentence on page 6, lines 127 to 129 should be eliminated or rewritten; otherwise this passage is confusing and falling out of context. The data used to perform the statistical analysis are not explained. So, the meaning of those data remains unclear for a reader without specific background knowledge of DTIA and/or VOH technology. I recommend you to add a short paragraph in Material & Method section (or to add an explaining subtext to the tables).

*Thank you for your kind notice. The sentence was omitted, and changes were made as you suggested. In the MM section we added the meaning of all DTIA and VOH variables, and our statistician author modified the paragraph explaining the statistical analysis.*

Reviewer #2:

- Dear authors, your revision pushed the manuscript into a more readable and understandable form/ structure. However, it is lacking sufficient scientific English, especially the punctuation and construction of sentences should be revised by a native speaker. Basically, there should be one sentence for one thought/statement. Some recommendations were given by me within the word file.

*Thank you very much for this comment. The manuscript was revised by a professional agency for academic editing. We are adding the proof as a supplementary file.*

-Since your data evaluation follows one possible approach and the raw data are not submitted within the supplement, I expect that you resubmit a SPSS or CSV or at least XLS file with the data set from the 228 images and all variables.

*During the first submission we submitted the XLS file and SPSS data in the supplementary files as directed by the journal policy. We will do it again in case they are missing.*

-I suggest a major revision, because the manuscript should be ordered as it is the standard. I commented within the word file which passages should be shifted.

*Thank you very much for your kindness. We followed the recommendations you inserted within the word file and made changes accordingly.*

*We also implemented the corrections and suggestions you made within the word file*

-Finally, the discussion should be revisited and more balanced between your novel findings and the established standard (VHO). This encompasses the reflection of absolute and relative changes despite of the p-value hype.

*As you pointed out, analyzing masticatory efficiency in complete denture wearers is the worst case scenario, and there are not many references using the colorimetric method that investigated ME in CDs wearers. We correlated the studies we analyzed to our findings and broadened the discussion in that way.*

-Please restructure your abstract according to the paper and your mentioned aims (i-iii), give the variables first and thereafter the results and conclusion.

*Thank you for this suggestion. We have structured the abstract in more proper way.*

- Please describe how this was done – it is important for the reproducibility of your study.

*The M&M section was rewritten, thanks to your comment. However, the methodology and procedure for the preparation of gum samples were adopted from the literature and we cited it properly for possible reproducibility.*

-Please give the software and operation, as well as if adjustments were set for this conversion.

*Thank you for this comment. It was a mistake not providing proper software details. After consulting the author KR who performed the DTIA and VOH, we made some modifications to the text as you suggested. There were no extra manual adjustments of the images, and she relied only on the software to automatically perform the analyses. That way we excluded any manual intrusion in the process.*

-Please add for each variable what a clinical relevant absolute or relative change would be.

*Since this was our first DTIA using and following the references regarding the method (there were not many), we decided to follow the changes of the uniformity, contract, entropy and homogeneity. Like other authors, we were not able to give exact values for each of them, but rather to discriminate them during the follow-ups. Of course, implementing the DTIA in dentate subjects might provide more insight into the absolute and relative change. That is our plan for future studies. As far as our edentulous subjects are concerned, for now DTIA has proven to be reliable, providing more detailed analyses than VOH.*

-Please describe how you handled the data from both sides of the specimen from both specimens per patient and point in time. This should be described in the subheadings below as well. If you decided for one side and one specimen only – describe why and how.

*Thank you for pointing to us this unclearness. Our statistician, author BM reorganized the section and wrote it in a more understandable way.*

-According to STROBE, you should give a flowchart on how many patients were screened and how many dropped out/ were closed out to which reasons. This is necessary to understand a potential selection bias.

*Thank you for this comment. We provided another flowchart.*

*-*The flowchart should start above your 19 patients.

*Yes, we created it accordingly. After a detailed review of our initial XLS data, and SPSS data, we, however, noticed that 18 participants were involved in the study and changed that throughout the text. Our colleague also made changes to Table 1, as requested.*

*-*Please contrast your overall findings from the DTIA with the published standard VOH. You can detect weaknesses and strength in both approaches. This should be described

*Thank you very much for this suggestion and raising that issue. We discussed the strength and weakness of DTIA at the beginning of the discussion. However, the colorimetric analysis is well established in the reference and we didn`t find ourselves competent to discuss it more. We added more sentences concerning the findings of DTIA and VOH.*

*The Clinical implications section was rewritten as you suggested. Thank you for pointing out.*

-No: you hypothesized it - you can draw the conclusion with the reasoning. This is…

*Thank you for your great help in formulating the conclusions. The whole section has been changed and we hope it has quality now and that the conclusions are drawn based on the presented data.*
